# Targeting mTOR in Glioblastoma: Rationale and Preclinical/Clinical Evidence

**DOI:** 10.1155/2018/9230479

**Published:** 2018-12-18

**Authors:** Carmen Mecca, Ileana Giambanco, Rosario Donato, Cataldo Arcuri

**Affiliations:** ^1^Department of Experimental Medicine, University of Perugia, Piazza Lucio Severi 1, 06132 Perugia, Italy; ^2^Centro Universitario per la Ricerca sulla Genomica Funzionale, Perugia Medical School, University of Perugia, Piazza Lucio Severi 1, 06132 Perugia, Italy

## Abstract

The mechanistic target of rapamycin (mTOR) drives several physiologic and pathologic cellular processes and is frequently deregulated in different types of tumors, including glioblastoma (GBM). Despite recent advancements in understanding the molecular mechanisms involved in GBM biology, the survival rates of this tumor are still disappointing, primarily due to the lack of efficacious treatments. The phosphatase and tensin homolog (PTEN)/phosphatidylinositol-3-kinase (PI3K)/protein kinase B (AKT)/mTOR pathway has emerged as a crucial player in GBM development and progression. However, to date, all the attempts to target this pathway with PI3K, AKT, or mTORC1 inhibitors failed to improve the outcome of patients with GBM. Despite these discouraging results, recent evidence pointed out that the blockade of mTORC2 might provide a useful therapeutic strategy for GBM, with the potential to overcome the limitations that mTORC1 inhibitors have shown so far. In this review, we analyzed the rationale of targeting mTOR in GBM and the available preclinical and clinical evidence supporting the choice of this therapeutic approach, highlighting the different roles of mTORC1 and mTORC2 in GBM biology.

## 1. Introduction

In the last decades, we witnessed important advancements in understanding the molecular mechanisms involved in GBM biology; however, GBM remains one of the deadliest types of tumor worldwide [1]. Indeed, the paradigm of medical treatment of chemotherapy plus radiation therapy has reached an efficacy plateau and several drugs designed to target one of the most deregulated pathways in GBM (PTEN/PI3K/AKT/mTOR) failed to improve the outcome of these patients [2–4]. More in detail, the limited blood-brain barrier penetration and the compensatory activation of collateral signaling pathways caused the failure of PI3K inhibitors [1, 4]. Additionally, the lack of mTORC2 inhibition that results in AKT activation led to disappointing results of mTORC1 inhibitors in the clinical trials conducted to date [5, 6]. On the other hand, emerging preclinical evidence suggests that targeting mTORC2 might provide an efficacious therapeutic strategy for GBM as it can overcome the limitations of mTORC1 inhibitors and pave the way for a personalized targeted treatment.

## 2. mTOR: A Brief Overview

### 2.1. mTORC1 Composition, Upstream Activators, and Downstream Targets

**Figure 1:**
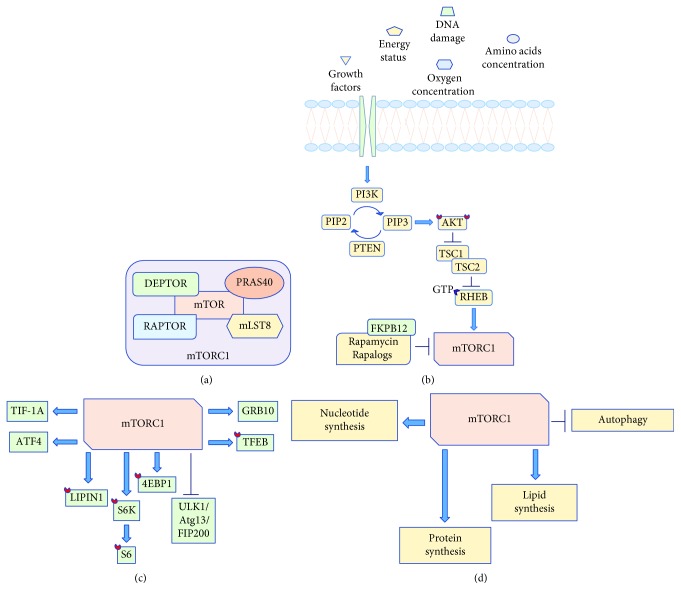
(a) mTORC1 protein composition; (b) mTORC1 upstream activators; (c) mTORC1 downstream targets; (d) mTORC1 cellular functions. Legend: DEPTOR: DEP domain TOR-binding protein; mTOR: mechanistic target of rapamycin; RAPTOR: regulatory-associated protein of mTOR; PRAS40: proline-rich AKT substrate 40 kDa; mLST8: mammalian lethal with Sec-13 protein 8; PI3K: phosphatidylinositol-3-kinase; PIP2: phosphatidylinositol (4,5)-bisphosphate; PIP3: phosphatidylinositol (3,4,5)-trisphosphate; PTEN: phosphatase and tensin homolog; AKT: protein kinase B; TSC1/2: tuberous sclerosis complex 1/2; GTP: guanosine triphosphate; RHEB: RAS homolog enriched in brain; FKBP12: 12-kDa intracellular FK506-binding protein; rapalogs: rapamycin analogs; 4EBP1: eukaryotic initiation factor 4E (eIF4E)-binding protein; S6K: ribosomal S6 kinases; GRB10: growth factor receptor-bound protein 10; ULK1/Atg13/FIP200: unc-51-like kinase 1/mammalian autophagy-related gene 13/focal adhesion kinase family-interacting protein of 200 kDa; ATF4: activating transcription factor 4; TFEB: transcription factor EB.

mTOR is a 289 kDa serine/threonine protein kinase localized in two structurally and functionally distinct multiprotein complexes known as mTORC1 and mTORC2 [7]. mTORC1 is composed of regulatory-associated protein of mTOR (RAPTOR), proline-rich AKT substrate 40 kDa (PRAS40), mammalian lethal with Sec-13 protein 8 (mLST8) and DEP domain TOR-binding protein (DEPTOR), and it is inhibited by rapamycin, a macrolide produced by the bacterium Streptomyces hygroscopicus (Figure 1(a)).

Rapamycin inhibits mTORC1 by binding the 12 kDa intracellular FK506-binding protein (FKBP12) that in turn directly interacts with mTORC1 but not with mTORC2 [7]. mTORC1 is activated by at least five cues: growth factors, stress, energy status, oxygen, and amino acid concentration. Growth factors, low energy status, low oxygen level, and DNA damage converge on the tuberous sclerosis complex 1/2 (TSC1/2) that acts as a GTPase-activating protein for the GTPase RAS homolog enriched in brain (RHEB), which in turn directly binds mTORC1 resulting into the stimulation of its kinase activity [7] (Figure 1(b)).

Upon activation, mTORC1 promotes cell growth by phosphorylating two downstream targets, namely, eukaryotic translation initiation factor 4E- (eiF4E-) binding protein 1 and ribosomal protein S6 kinase (S6K) that drive protein synthesis (Figures 1(c) and 1(d)). Indeed, S6K phosphorylates the 40S ribosomal subunit, thus triggering the translation of mRNA transcripts with 5′-terminal oligopolypyrimidine, and phosphorylates the eukaryotic translation initiation factor (eIF)4B on serine 422, ultimately promoting eIF4B association with eIF3 and eIF4F complex formation. On the other hand, mTORC1-mediated phosphorylation of 4EBP1 causes the release of eIF4E from 4EBP1, allowing eIF4E-eIF4G association and cap-dependent translation [7]. Furthermore, mTORC1 contributes to protein synthesis by activating the transcription intermediary factor 1-alpha (TIF-1A) that induces RNA polimerase to transcribe rRNA genes and also by inhibiting a polimerase III repressor known as MAF1, thus enabling 5sRNA and tRNA transcription [8, 9] (Figure 1(c)). Another mTORC1 target is the growth factor receptor-bound protein 10 (GRB10), whose activation is responsible for the degradation of the insulin receptor substrate-1 (IRS-1) and the feedback inhibition of PI3K [10] (Figure 1(c)). In addition to its extensively investigated role in protein synthesis, mTORC1 also participates in lipid and nucleotide synthesis, whose rapid turnover is a hallmark of tumors, including GBM (Figure 1(d)). More in detail, mTORC1 phosphorylates LIPIN-1 and prevents it from entering the nucleus, resulting in the suppression of the sterol regulatory element-binding protein 1/2 (SREBP1/2), a transcription factor involved in fatty acid and cholesterol synthesis [11] (Figure 1(c)). Instead, mTORC1 contribution to purine synthesis occurs through the induction of the activating transcription factor 4 (ATF4) that in turn triggers methylenetetrahydrofolate dehydrogenase (NADP+ dependent) 2, methenyltetrahydrofolate cyclohydrolase (MTHFD2) expression, a key component of the mitochondrial tetrahydrofolate cycle [12] (Figure 1(c)). Lastly, mTORC1 contributes to tumor cell growth by inhibiting autophagy, a catabolic pathway that degrades aged or damaged organelles (Figure 1(d)); mTORC1 suppresses autophagy directly by inhibiting the kinase complex unc-51-like kinase 1/mammalian autophagy-related gene 13/focal adhesion kinase family-interacting protein of 200 kDa (ULK1/Atg13/FIP200) which is a key component required for the autophagy induction and indirectly by modulating the expression of death-associated protein 1 (DAP1), a novel substrate of mTORC1 that negatively regulates autophagy [13, 14] (Figure 1(c)). Along with the aforementioned mechanisms, mTORC1 also blocks autophagy induction through the negative regulation of lysosome biogenesis; indeed, mTORC1 can inhibit the expression of genes involved in lysosomal functions by phosphorylating the transcription factor EB (TFEB) and preventing its nuclear entry [15, 16] (Figure 1(c)).

### 2.2. mTORC2 Composition, Upstream Activators, and Downstream Targets

**Figure 2:**
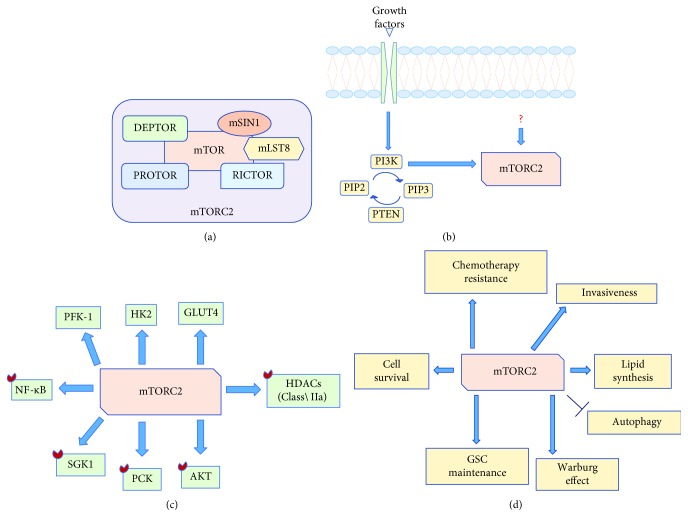
(a) mTORC2 protein composition; (b) mTORC2 upstream activators; (c) mTORC2 downstream targets; (d) mTORC2 cellular functions. Legend: mTOR: mechanistic target of rapamycin; RICTOR: rapamycin-insensitive companion of mTOR; mLST8: mammalian lethal with Sec-13 protein 8; DEPTOR: DEP domain TOR-binding protein; PROTOR: protein observed with RICTOR; mSIN1: stress-activated map kinase-interacting protein 1; PI3K: phosphatidylinositol-3-kinase; PIP2: phosphatidylinositol (4,5)-bisphosphate; PIP3: phosphatidylinositol (3,4,5)-trisphosphate; PTEN: phosphatase and tensin homolog; AKT: protein kinase B; SGK1: serum and glucocorticoid-regulated kinase 1; PKC: protein kinase C; HDACs: histone deacetylases; PFK-1: phosphofruttokinase-1; HK2: hexokinase 2; GLUT4: glucose transporter type 4; NF-*κ*B: nuclear factor kappa-light-chain-enhancer of activated B cells.

Differently, mTORC2 is composed of the rapamycin-insensitive companion of mTOR (RICTOR), DEPTOR, mLST8, stress-activated map kinase-interacting protein 1 (mSIN1), and protein observed with RICTOR (PROTOR) and is considered rapamycin insensitive because, as previously mentioned, the complex rapamycin-FKBP12 is not able to directly bind mTORC2 [7] (Figure 2(a)).

However, it has been demonstrated that prolonged treatment with rapamycin might inhibit mTORC2 assembly by sequestering mTOR in some cell cultures [17]. Differently from mTORC1, less is known about mTORC2 upstream activators; it is triggered by growth factors but does not respond to nutrients [7] (Figure 2(b)). Once activated, mTORC2 drives cell proliferation, motility, and survival primarily through the activation of different AGC protein kinases (Figure 2(c)). In fact, mTORC2 phosphorylates protein kinase C (PKC)*δ*, PKC*ζ*, PCK*γ*, and PKC*ε* that are involved in cytoskeleton assembly and cell migration, besides AKT on serine 473 [18–21] (Figure 2(c)). Intriguingly, it has recently been demonstrated that mTORC2 triggers the activation of the serum and glucocorticoid-regulated kinase 1 (SGK1), which is involved in ion transport and cell survival [22] (Figures 2(c) and 2(d)).

The direct involvement of mTORC2 in GBM biology clearly emerged in a Drosophila glioma model obtained by hyperactivating the epidermal growth factor receptor (EGFR), RAS and PI3K. In this model, it has been observed that RICTOR and mSIN1 loss of function prevented tumor formation [23]. Later on, Bashir et al. established that RICTOR overexpression alone was sufficient to promote multifocal infiltrating oligodendroglial tumors in the subventricular zone and lateral ventricles of mice that showed an increased mTORC2 activity that sustained cancer stem cell amplification [24]. In addition to the role in tumor induction, mTORC2 activation is also responsible for GBM growth and progression. Indeed, Gulati el al. observed that treatment of GBM cell lines with rapamycin not only resulted in a time-dependent decrease of S6K phosphorylation but also caused a paradoxical increase of AKT phosphorylation on serine 473 which is known to be responsible for cell proliferation. The same authors reported that this paradoxical increase of AKT phosphorylation can be reversed by RICTOR but not RAPTOR knockdown by siRNA [25].

mTORC2 is also involved in the induction of the Warburg effect, a metabolic process by which tumor cells metabolize glucose via the aerobic glycolysis also in the presence of sufficient oxygen levels to supply the macromolecular demand of rapidly growing cells (Figure 2(d)). Indeed, by stimulating AKT phosphorylation on serine 473, mTORC2 triggers the expression of the glucose transporter type 4 (GLUT4) and the activation of the glycolytic enzyme hexokinase 2 (HK2) and phosphofruttokinase-1 (PFK-1) [26–28] (Figure 2(c)). Moreover, mTORC2 phosphorylates and inactivates class IIa histone deacetylases (HDACs) that causes Forkhead box O (FOXO)1 and FOXO3 acetylation, resulting in c-MYC release from the suppressive miR-34-c [29] (Figure 2(c)). More recently, it has been shown that the acetyl coenzyme A (acetyl-CoA) derived from glucose and lactate metabolism is used by GBM cells to induce RICTOR acetylation that results in mTORC2 activation; this mechanism creates an autoactivation loop by which mTORC2 triggers cell proliferation and growth, bypassing growth factor-activated upstream signaling and rendering GBM cells resistant to receptor tyrosine kinase inhibitors [30]. Additionally, mTORC2 has recently been reported to confer resistance to the alkylating agent cisplatin via nuclear factor kappa-light-chain-enhancer of activated B cell (NF-*κ*B) activation in an AKT-independent way [31] (Figures 2(c) and 2(d)). In this study, Tanaka et al. demonstrated that NF-*κ*B signaling is upregulated in GBM cell lines and in GBM patients treated with rapamycin and that the inhibition of both mTORC1 and mTORC2 with the mTOR kinase inhibitor PP242 sensitizes *EGFRvIII-*mutant tumors to cisplatin-induced cell death, confirming the direct involvement of mTORC2 activation in chemotherapy resistance [31]. As previously mentioned with regard to mTORC1, mTORC2 also participates in lipid synthesis (Figure 2(d)). In GBM, the cleavage of SREBP1 that occurs on Golgi membranes and is triggered by mutant *EGFR* has been demonstrated to be rapamycin insensitive. By contrast, mTORC2 induces SREBP1 cleavage through AKT-dependent and AKT-independent mechanisms, resulting in the expression of acetyl-CoA carboxylase, fatty acid synthase and acyl-CoA synthetase genes, which are all involved in fatty acid and cholesterol synthesis [32, 33].

## 3. Preclinical Data and Clinical Application of mTOR Kinase Inhibitors

Despite the pivotal role of mTOR in GBM which is now widely recognized, the first generation of mTORC1 inhibitors gave disappointing results in clinical trials. The magnitude of this failure is ascribable to the pharmacodynamic properties of these compounds, as they only target mTORC1, leading to an incomplete inhibition of mTORC1 downstream targets and to the deregulation of a negative feedback following mTORC1 inhibition that results in PI3K reactivation [10]. In addition, the lack of activity against mTORC2 represents another major clinical limitation to the efficacy of rapamycin analogs (rapalogs) [34]. To overcome these limitations, a new generation of ATP-competitive mTOR kinase inhibitors has been developed. These compounds include Torin1, PP242, PP30, Ku-0063794 (KuDOS Pharmaceuticals), WAY-600 (Wyeth), WYE-687 (Wyeth), WYE-354 (Wyeth), INK128 (Intellikine), CC214-1/2 (Celgene Corporation, San Diego, U.S.A.), AZD2014, AZD8855 (AstraZeneca), and OSI-027 (OSI Pharmaceuticals) and have been designed in order to target the mTOR kinase domain and irreversibly block both mTORC1 and mTORC2 activation [35]. Some of these compounds have been tested both *in vitro* and *in vivo* and confirmed the pivotal role of mTORC2 in GBM biology. Indeed, Gini et al. proved that the mTOR kinase inhibitors CC214-1and CC214-2 (orally available) (Celgene Corporation (San Diego, U.S.A.)) are able to overcome the limitations of rapamycin and rapalogs and to inhibit GBM growth by blocking mTORC2 activity *in vitro* and *in vivo*, respectively [36, 37]; moreover, the same authors demonstrated that the sensitivity to CC214 compounds is significantly increased in the presence of EGFRvIII and PTEN loss and that the pharmacologic inhibition of autophagy induced by CC214 sensitizes GBM cells to cell death, preventing a cytostatic effect [37]. Furthermore, Kahn et al. demonstrated that AZD2014 enhances the radiosensitivity of glioblastoma stem cells (GSCs) *in vitro* and under *in vivo* orthotopic conditions by inhibiting mTORC1/2 [38]. Additionally, our group has recently demonstrated that the treatment of genetically different GBM cell lines with PP242 but not with rapamycin induces a dramatic and permanent reduction of AKT phosphorylation on serine 473 that not only counteracts tumor growth and invasiveness but also prevents GSC proliferation. Moreover, we also proved that mTORC2 activation is independent from PI3K, as the irreversible inhibition of PI3K with wortmannin is not able to prevent mTORC2 activation, which is evaluable analyzing mTOR phosphorylation on serine 2481 [39].

**Figure 3:**
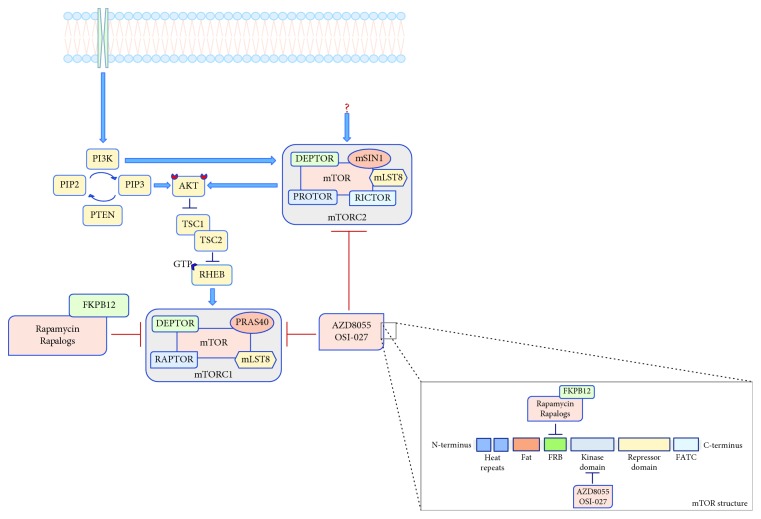
Schematic representation showing paths and points of action of the mTOR kinase domain inhibitors (AZD8055 (AstraZeneca) and OSI-027 (OSI Pharmaceuticals)) currently evaluated in clinical trials. Legend: PI3K: phosphatidylinositol-3-kinase; PIP2: phosphatidylinositol (4,5)-bisphosphate; PIP3: phosphatidylinositol (3,4,5)-trisphosphate; PTEN: phosphatase and tensin homolog; AKT: protein kinase B; TSC1/2: tuberous sclerosis complex 1/2; GTP: guanosine triphosphate; RHEB: RAS homolog enriched in brain; mTOR: mechanistic target of rapamycin; RAPTOR: regulatory-associated protein of mTOR; PRAS40: proline-rich AKT substrate 40 kDa; mLST8: mammalian lethal with Sec-13 protein 8; DEPTOR: DEP domain TOR-binding protein; RICTOR: rapamycin-insensitive companion of mTOR; PROTOR: protein observed with RICTOR; mSIN1: stress-activated map kinase-interacting protein 1; FKBP12: 12-kDa intracellular FK506-binding protein; rapalogs: rapamycin analogs; FATC: FAT carboxyl terminal; FRB: FKBP-rapamycin-binding domain.

As preclinical studies confirmed the superiority of mTOR kinase inhibitors compared to rapalogs and have revealed the efficacy of mTORC2 inhibition in counteracting GBM growth, invasiveness, and GSC proliferation, the mTOR kinase inhibitors AZD8055 (AstraZeneca) and OSI-027 (OSI Pharmaceuticals) have already entered clinical trials [40] (Figure 3). A phase I study of AZD8055 (AstraZeneca) in advanced solid malignancies (NCT00973076) and in recurrent GBM (NCT01316809) has completed the recruitment, and results are eagerly awaited; a phase I study of OSI-027 (OSI Pharmaceuticals) in advanced solid tumors and lymphoma started in 2008 and is now completed (NCT00698243). As single agent, OSI-27 has shown to be well tolerated and evidence of activity has emerged [41].

## 4. Evaluation of mTOR Activation in GBM Patients

Despite PTEN/PI3K/AKT/mTOR pathway is considered a hallmark of GBM and the inhibition of this pathway represents to date an interesting strategy against this lethal tumor [1, 42], the direct evaluation of this pathway activation in GBM patients is not routinely performed or standardized and results are still controversial. In this context, the first analysis of PTEN/PI3K/AKT/mTOR pathway activation in patient specimens was carried out on 45 untreated primary GBM; immunohistochemistry analysis revealed that PTEN loss correlated with AKT activation and that in turn AKT phosphorylation significantly correlated with mTOR, FOXO1, FOXO3a, FOXO4, and S6 activation [43]. Moreover, a tight relation between EGFRvIII expression and the activation of PI3K downstream targets has been also observed in this study [43] (Table 1).

Later on, Chakravarti et al. analyzed the expression of total PI3K, AKT, and S6K in 92 gliomas with different malignancy degree by western blot and did not find any difference in the total expression of PI3K, AKT, and S6K between GBM and non-GBM tumors. However, despite the total expression of these protein kinases was unchanged between groups, the authors reported that PI3K, AKT, and S6K phosphorylation was significantly higher in GBM versus non-GBM tumors. Moreover, the levels of PI3K, AKT, and S6K phosphorylation were inversely related to the expression of the cleaved caspase 3 and correlated with radiation resistance and an adverse outcome [44] (Table 1). The activation status of AKT and in addition of NF-*κ*B and STAT3 and their correlation with tumor grade has also been analyzed in 259 diffuse gliomas by Wang et al., by microarray and immunohistochemistry. These authors described consistent AKT and NF-*κ*B activation in tumor samples but not in astrocytes or oligodendrocytes of normal brain cortex and cerebellum; moreover, these authors reported a positive correlation between AKT, NF-*κ*B activation, and tumor grade but not between them and STAT3 [45] (Table 1). In another study, Riemenschneider et al. reported the colocalization of TSC2, mTOR, 4EBP1, S6K, S6, and STAT3 phosphorylation with AKT activation, although only TSC2, S6K, and S6 phosphorylation has been found to correlate with AKT activation [46] (Table 1).

Contrariwise, Fiano et al. did not find any correlation between AKT phosphorylation, cyclin D1, p27/Kip1, and *PTEN* or *EGFR* mutations in 65 GBM surgical samples [47]. Instead, Hlobilkova et al. found a strong relation between EGFR expression and tumor grade in 89 samples of glioma with different malignancy degree but they observed comparable levels of AKT phosphorylation between low- and high-grade gliomas [48] (Table 1).

Higher levels of AKT, mTOR, and S6K phosphorylation in high-grade glioma compared with low-grade glioma have been also reported by Li et al., who analyzed 87 tissue samples and found that the percentage of patients with high AKT, mTOR, and S6K phosphorylation, as detected by immunohistochemistry, was greater in grades III and IV than in grades I and II glioma [49] (Table 1). Similarly, Korkolopoulou et al. analyzed the expression of mTOR, S6K, and 4EBP1 phosphorylation in 111 tissue samples (grades II–IV) by immunohistochemistry and validated their analysis in 3 primary GBM cell cultures by western blotting analysis [50] (Table 1). They found that while normal tissues had no positivity for all the proteins considered, none of the tumor samples was negative for mTOR, S6K, or 4EBP1 phosphorylation. With the exception of one sample, tissues that showed mTOR positivity were also positive for S6K staining and all mTOR-positive specimens also showed 4EPB1 coexpression. Moreover, these authors demonstrated that 4EBP1 phosphorylation increased with the histological grade and that mTOR phosphorylation was higher in grade III/IV glioma compared with grade II. Of note, no significant difference in terms of S6K phosphorylation was reported according to tumor grades. They next sought to investigate the correlation between the level of mTOR/S6K/4EBP1 phosphorylation and survival and found that 4EBP1 expression was an independent adverse prognostic index in all the cohort analyzed, while the increased level of mTOR phosphorylation correlated with disease-free survival (DFS) [50] (Table 1). More recently, Machado et al. demonstrated that mTOR expression was significantly higher in wild-type *IDH1* primary GBM, when compared to healthy tissue, and was also higher compared with *R132H IDH1*-mutant GBM [51]. In addition, these same authors proved that mTOR phosphorylation on serine 2448 and S6 phosphorylation on serine 240 and 244 were increased in wild-type *IDH1* GBM compared with *R132H IDH1*-mutant GBM [51] (Table 1).

Compared with mTORC1 and its upstream and downstream targets, the evaluation of mTORC2 activation in GBM patients is further underestimated. The activation of mTORC2 has been evaluated in 5 GBM cell lines, in 31 tumor samples and 5 normal brain tissues by Masri et al. [52]. These authors observed that the expression of activated mTORC2 was quite undetectable in normal brain tissue while it was high in the tumor cell lines they analyzed. Consistently, they observed increased RICTOR expression and extent of AKT phosphorylation on serine 473 only in tumor cells. Of note, RICTOR overexpression appeared to be independent of *PTEN* status. In accordance with the *in vitro* data, these same authors found that the 86% of tumor samples had RICTOR overexpression and 70% of them showed high mTORC2 activity [52] (Table 1). More recently, Alvarenga et al. described RICTOR expression and AKT phosphorylation on serine 473 in 195 patients with brain tumors (38 grade I, 49 grade II, 15 grade III, and 93 grade IV astrocytoma) and correlated AKT activation with overall survival (OS) [53]. They did not find any differences in AKT phosphorylation on serine 473 between low-grade glioma and normal brain tissue but they observed a significant increase in AKT phosphorylation in GBM patients compared with normal brain tissue; moreover, the increased expression of activated AKT correlated with a reduced OS [46]. These same authors analyzed RICTOR expression, and although they did not find an increased expression of this mTORC2 component between normal brain tissue and all grade astrocytoma, they observed nuclear localization of RICTOR in GBM that might suggest a change of its binding partner and a possible implication in tumor progression [53] (Table 1).

## 5. Discussion and Future Perspective

As discussed, mTOR pathway is certainly one of the most compelling mechanisms driving GBM biology. However, to date, there are still some cruxes that need to be unraveled to translate the encouraging preclinical results reported in the clinical management of GBM patients.

First of all, the direct evaluation of mTOR pathway activation in GBM patients is not routinely performed and results are affected by discrepancies due to different methodologies of quantification applied by distinct laboratories, which makes it challenging to select those patients that, based on their molecular profile, might mainly benefit of treatment with mTOR kinase inhibitors. As it is now widely accepted that genetic background influences and predicts the outcome of targeted therapy, one of the future challenges for GBM treatment will be to improve the precision and reproducibility of molecular analysis of mTOR pathway activation and standardize this evaluation among laboratories.

Another concern regarding the use of mTOR kinase inhibitors in clinic is their potential immunosuppressive activity, as the impairment of the immune system is widely known to favor tumor growth and progression. However, assays of adaptive immune functions of 1–3 weeks in leukemia revealed that the anticancer properties of PP242 are dominant over its immunosuppressive activity compared with those of rapamycin and another mTOR kinase inhibitor known as PI103 but the outcome of prolonged treatment with this compound and other mTOR kinase inhibitors remains to be investigated [54]. Additionally, as most of the clinical trials evaluating mTOR kinase inhibitors in GBM are ongoing, data regarding the tolerability and the safety profile of these drugs are still not available and the results of these studies will further help to define the role of mTOR as therapeutic target, hopefully providing a new tool against GBM.

## Figures and Tables

**Table 1:** Clinical evaluation of PTEN/PI3K/AKT/mTOR pathway activation in GBM patients.

No. of samples	Methods	Main findings	Reference
45	Immunohistochemistry	Correlation between PTEN loss and AKT activation, correlation between AKT phosphorylation and FOXO and S6 activation, and correlation between EGFRvIII expression and PI3K pathway activation	43
92	Western blot	No difference in PI3K AKT and S6K total expression between GBM and non-GBM tumors; increased PI3K, AKT, and S6K phosphorylation in GBM compared with non-GBM tumors; correlation between PI3K, AKT, and S6K phosphorylation and adverse outcome	44
259	Microarray and immunohistochemistry	AKT and NF-*κ*B activation in tumor samples and not in normal brain and positive correlation between AKT and NF-*κ*B activation and tumor grade	45
29	Immunohistochemistry	Correlation between AKT activation and TSC2, S6K, and S6 phosphorylation	46
65	Western blot and immunohistochemistry	No correlation between AKT activation and EGFR or PTEN status	47
89	Immunohistochemistry	No difference of AKT phosphorylation between low- and high-grade glioma	48
87	Immunohistochemistry	Higher levels of AKT, mTOR, and S6K in high-grade glioma compared with low-grade glioma	49
111 + 3 primary GBM cell cultures	Immunohistochemistry and western blot	Absence of mTOR, S6K, and 4EBP1 positivity in normal brain tissues; increase of mTOR and 4EBP1 phosphorylation with histological grade; correlation between 4EBP1 expression and adverse prognosis; correlation between mTOR phosphorylation and disease-free survival	50
225	Immunohistochemistry	Higher mTOR expression in WT *IDH1* GBM compared with healthy tissues and *R132H IDH1*-mutant GBM and increased mTOR and S6 phosphorylation in WT *IDH1* GBM compared with *R132H IDH1*-mutant GBM	51
36 + 5 GBM cell cultures	Immunohistochemistry, western blot, real-time PCR, and cell proliferation and migration assays	Undetectable mTORC2 activation in normal cells and high expression in GBM cell cultures and correlation between RICTOR expression and mTORC2 activation	52
196	Immunohistochemistry and western blot	Increased AKT phosphorylation in GBM compared with normal brain tissues and correlation between increased AKT activation and reduced overall survival	53

## Abbreviations

mTOR: Mechanistic target of rapamycin
GBM: Glioblastoma
PTEN: Phosphatase and tensin homolog
PI3K: Phosphatidylinositol-3-kinase
AKT: Protein kinase B
RAPTOR: Regulatory-associated protein of mTOR
PRAS40: Proline-rich AKT substrate 40 kDa
mLST8: Mammalian lethal with Sec-13 protein 8
DEPTOR: DEP domain TOR-binding protein
FKBP12: 12 kDa intracellular FK506-binding protein
TSC1/2: Tuberous sclerosis complex 1/2
RHEB: RAS homolog enriched in brain
S6K: Ribosomal S6 kinase
4EBP1: Eukaryotic initiation factor 4E- (eIF4E-) binding protein
eIF: Eukaryotic translation initiation factor
TIF-1A: Transcription intermediary factor 1-alpha
GRB10: Growth factor receptor-bound protein 10
IRS-1: Insulin receptor substrate-1
SREBP1/2: Sterol regulatory element-binding protein 1/2
ATF4: Activating transcription factor 4
MTHFD2: Methylenetetrahydrofolate dehydrogenase (NADP+ dependent) 2, methenyltetrahydrofolate cyclohydrolase
ULK1/Atg13/FIP200: Unc-51-like kinase 1/mammalian autophagy-related gene 13/focal adhesion kinase family-interacting protein of 200 kDa
DAP1: Death-associated protein 1
TFEB: Transcription factor EB
RICTOR: Rapamycin-insensitive companion of mTOR
mSIN1: Stress-activated map kinase-interacting protein 1
PROTOR: Protein observed with RICTOR
PKC: Protein kinase C
SGK1: Serum and glucocorticoid-regulated kinase 1
EGFR: Epidermal growth factor receptor
GLT4: Glucose transporter type 4
HK2: Hexokinase 2
PFK-1: Phosphofruttokinase-1
HDACs: Histone deacetylases
FOXO: Forkhead box O
Acetyl-CoA: Acetyl coenzyme A
NF-*κ*B: Nuclear factor kappa-light-chain-enhancer of activated B cells
Rapalogs: Rapamycin analogs
GSC: Glioblastoma stem cell
DFS: Disease-free survival
OS: Overall survival.
